# Expanding the spectrum of non-canonical NUT carcinoma: clinicopathological and molecular characterization of *BRD3::NUTM1* and *WWTR1::NUTM1* fusion variants

**DOI:** 10.3389/fonc.2026.1828484

**Published:** 2026-07-01

**Authors:** Zuoyu Liang, Chan Yang, Xiaoqian Zhai, Yan Wang, Min Chen, Mei Tang, Linlin Huang, Mengqian Li, Ping Zhou, Xuexue Wu, Ji Bao, Dongni Liang, Lili Jiang, Weiya Wang

**Affiliations:** 1 Department of Pathology of West China Hospital, Sichuan University, Chengdu, China; 2 Department of Pathology of West China Tianfu Hospital, Sichuan University, Chengdu, China; 3 Lung Cancer Center and Institute of West China Hospital, Sichuan University, Chengdu, China; 4 Pathological Diagnosis Center, Sichuan Kingmed Center for Clinical Laboratory Co., Ltd., Chengdu, China; 5 Institute of Clinical Pathology of West China Hospital, Sichuan University, Chengdu, China; 6 Department of Pathology, West China Second University Hospital, Sichuan University, Chengdu, China; 7 Key Laboratory of Birth Defects and Related Diseases of Women and Children (Sichuan University), Ministry of Education, Chengdu, China

**Keywords:** *BRD3*, gene fusion, nuclear protein in testis (NUT) carcinoma, *NUTM1*, *WWTR1*

## Abstract

**Introduction:**

Nuclear protein in testis (NUT) carcinoma (NC) is an extremely rare and highly aggressive tumor. Non-canonical NC patients frequently have unique clinicopathological characteristics in terms of location, molecular alteration, and outcome, with morphological overlap with other tumors. Here, we report and summarize the non-canonical clinicopathological features of three unusual NC patients harboring *BRD3::NUTM1* or *WWTR1::NUTM1* gene rearrangement.

**Methods:**

The data of three patients with NC who were diagnosed between 2019 and 2022 at the Department of Pathology at West China Hospital or the Sichuan Kingmed Center for Clinical Laboratory were collected. Immunohistochemistry (IHC), fluorescence *in situ* hybridization (FISH), and RNA sequencing were performed. A literature review was conducted for NC patients with non-canonical features.

**Results:**

All three NC patients were male and aged between 31 and 60 years. The primary sites were the gastric corpus, nasal cavity, and subcutaneous tissue of the calvaria vertex. Histologically, two patients harbored NC with glandular differentiation, and one harbored NC with small cell carcinoma-like morphology. IHC revealed positivity for Pan-CK (cytokeratin) (PCK) (3/3), Epithelial membrane antigen (EMA) (3/3), Cytokeratin 5/6 (CK5/6) (3/3), and P63 (2/3). The Ki-67 index ranged from 20% to 40%. The remaining IHC markers were negative. RNA sequencing revealed *BRD3::NUTM1* fusion in two patients and *WWTR1::NUTM1* fusion in one patient.

**Conclusion:**

In our study, the clinicopathological features of NC in patients with rare molecular alterations are reported and summarized. As increasing numbers of *NUTM1*-rearranged tumors with diverse histologic phenotypes have been reported, the traditional classification of NC as a purely epithelial malignancy may warrant reconsideration. Non-canonical NC needs to be further reported, integrated, and classified to precisely guide targeted therapy.

## Introduction

Nuclear protein in testis (NUT) carcinoma (NC) is a rare, highly aggressive malignant tumor characterized by a fusion of the *NUTM1* gene (referred to as the *NUT* gene) on chromosome 15 with other partner genes, with the most common partner gene being *BRD4* (>70%). Although NC was initially reported in children and adolescents, it can affect individuals of any age with no gender predisposition. The predominant locations of NC are the head and neck, upper respiratory tract, and mediastinum. NC shows no clear histological specificity and mostly presents as poorly differentiated squamous cell carcinoma (SCC) (1, 2).

In this study, we evaluated three patients with non-*BRD4* fusion-type NC that occurred in the stomach, nasal cavity, and top of the head. Histologically, two patients showed poorly differentiated carcinomas with glandular differentiation, and one patient showed small cell carcinoma-like morphology. Notably, *BRD3::NUTM1* gene rearrangement was detected in one patient with glandular differentiation and in another with small cell carcinoma-like morphology, while *WWTR1::NUTM1* gene rearrangement was detected in a patient with glandular differentiation.

As attention to NC has increased, increasing numbers of NCs with non-*BRD4::NUTM1* fusion have been identified. These rare NC types often have non-canonical pathological manifestations and clinical processes (3–8), suggesting that non-*BRD4* fusion-type NCs have the potential to be considered a special tumor subtype. By reviewing the literature and evaluating three unusual NC patients, we aimed to describe the clinicopathological features of these rare non-canonical NC and further expand the recognized molecular and histological spectrum of this entity.

## Materials and methods

### Case selection and literature search

Formalin-fixed paraffin-embedded (FFPE) samples diagnosed as NC with unusual morphological features were collected. These cases were diagnosed between 2019 and 2022 at West China Hospital of Sichuan University and Sichuan Kingmed Center for Clinical Laboratory. The Ethics Committee on Biomedical Research, West China Hospital of Sichuan University, provided institutional authorization and approval (approval number: 1016) for this retrospective observational research. Written informed consent was obtained from the patient’s next of kin for the publication of any potentially identifiable images or data included in this article.

In this study, “non-canonical NC” is defined as *NUTM1*-rearranged tumors with fusion partners other than *BRD4*. These cases often exhibit significant differences in clinicopathological features and biological behavior compared to canonical NC. For the literature review of NC patients, the keyword “NUT carcinoma” was searched in PubMed from 2010 to 2024. **Table 1** further integrates non-canonical NC, excluding cases with canonical pathological features or incomplete clinical information (especially unclear fusion subtypes or incomplete prognosis).

**Table 1:** Summary of current reported cases of *NUT*-rearranged neoplasm with non-canonical clinicopathological features in the literature.

Case	Author	Gene fusion	Age (years)	Sex	Location	Histological feature	IHC positive	Treatment	Outcome (months)
Case 1	Current study	*BRD3::NUT* t(9;15)(q34;q14)	31	M	Stomach	Small cell-like	PCK, CK5/6, CK7, P63 EMA	None	Alive (5)
Case 2	Current study	*WWTR1::NUT* t(3;15)(q25;q14)	60	M	Calvaria vertex	Glandular differentiation	PCK, CK5/6, CK7, P63, EMA	Surgery and RCT	Dead (40)
Case 3	Current study	*BRD3::NUT* t(9;15)(q34;q14)	42	M	Nasal cavity	Glandular differentiation	PCK, CK5/6, CK7, EMA	RCT	Dead (5)
Case 4	Nishimura et al. (24)	*BRD3::NUT*	46	F	Inguinal subcutaneous mass	Glandular differentiation	CK5/6, CK7, P40	Surgery and CT	Alive (132)
Case 5	Lee et al. (50)	*BRD4::NUT*	5	M	Anterior medium	Glandular differentiation	CK, EMA, CEA	CT	Dead (1.5)
Case 6	Xu et al. (31)	*BRD4::NUT*	36	F	Kidney	Glandular differentiation	CK7, CK20, 34βE12	NA	Dead (6)
Case 7	Stevens et al. (3)	*BRD4::NUT*	44	M	Lung	Glandular differentiation	PCK, CK5/6, TTF-1, P63, P16	NA	NA
Case 8	Chien et al. (20)	*ZNF532::NUT*	21	F	Mandible bone	Rhabdoid	CD34	Surgery and RT	Alive (43)
Case 9	Dickson et al. (26)	*MXD1::NUT*	39	F	Stomach wall	Rhabdoid	CK, GFAP	Surgery and CT	Alive (108)
Case 10	Dickson et al. (26)	*BCORL1::NUT*	45	M	Arm	Pleomorphism	NA	Surgery and RCT	Dead (48)
Case 11	Schaefer et al. (25)	*CIC::NUT*	60	M	Masticator space	Myoepithelial-like	CK7, ETV4	CT and immunotherapy	Alive (10)
Case 12	Le Loarer et al. (4)	*CIC::NUT*	3	M	Temporal	Epithelioid	PCK, ETV4	Surgery and RCT	Dead (18)
Case 13	Le Loarer et al. (4)	*CIC::NUT*	5	M	Occipital	Epithelioid	KL1, EMA, P40, CD99, ETV4	CT	Dead (14)
Case 14	Le Loarer et al. (4)	*CIC::NUT*	7	F	Paravertebral	Epithelioid	ETV4, CD99, SOX-2	Surgery and CT	Dead (37)
Case 15	Le Loarer et al. (4)	*CIC::NUT*	27	M	Lung	Epithelioid	ETV4, WT-1, SOX-2	Surgery and CT	Dead (7)
Case 16	Le Loarer et al. (4)	*CIC::NUT*	22	F	Lateral ventricule	Epithelioid	PCK, CD99, ETV4	Surgery and RCT	Dead (17)
Case 17	Le Loarer et al. (4)	*CIC::NUT*	18	M	Epidural space	Epithelioid	WT-1	Surgery and RCT	Alive (40)
Case 18	Goto et al. (21)	*MGA::NUT*	49	M	Lung	Sarcoma-like	BCOR, bcl-2, CD99, Syn, neurofilament, MUC4	Surgery and RCT	Dead (13)
Case 19	Stevens et al. (3)	*MGA::NUT*	63	F	Lung	Sarcoma-like	NA	NA	NA
Case 20	Stevens et al. (3)	*MGA::NUT*	61	M	Chest wall/pleural	Sarcoma-like	CD34	NA	NA
Case 21	Diolaiti et al. (27)	*MGA::NUT*	10	M	Thigh	Sarcoma-like	CD99, CD34, bcl-2, desmin	Surgery and RCT	Alive (132)
Case 22	Diolaiti et al. (27)	*MGA::NUT*	10	F	Dura mater	Sarcoma-like	CD99, desmin	Surgery and RT	Alive (15)
Case 23	Wangsiricharoen et al. (5)	*MGA::NUT*	10	M	Thigh	Sarcoma-like	NA	Surgery	Alive (180)
Case 24	Wangsiricharoen et al. (5)	*MGA::NUT*	14	M	Chest well	Sarcoma-like	S-100, CD56, neurofilament, CD99, calponin, TLE1	Surgery	Alive (18)
Case 25	Wangsiricharoen et al. (5)	*MGA::NUT*	28	M	Pelvis	Sarcoma-like	CD56, CD99	Surgery	Dead (84)
Case 26	Underwood et al. (28)	*MGA::NUT*	48	M	Foot	Sarcoma-like	CD99	Surgery	Alive (6)
Case 27	Mantilla et al. (29)	*MGA::NUT*	61	M	Pleura	Sarcoma-like	CD34	NA	Alive (NA)
Case 28	Stevens et al. (3)	*MXD4::NUT*	65	F	Colon (cecum)	Sarcoma-like	Vimentin	NA	NA
Case 29	Van Treeck et al. (6)	*MXD4::NUT*	38	F	Sigmoid colon	Sarcoma-like	CD34, SMA	CT	Alive (15)
Case 30	Van Treeck et al. (6)	*MXD4::NUT*	40	M	Ileocecal valve	Sarcoma-like	SMA, ERG	NA	Alive (5)
Case 31	Van Treeck et al. (6)	*MXD4::NUT*	65	F	Cecum	Sarcoma-like	Syn	NA	Dead (30)
Case 32	Van Treeck et al. (6)	*MXD4::NUT*	44	F	Descending colon	Sarcoma-like	CD99	NA	Alive (10)
Case 33	Van Treeck et al. (6)	*MXD4::NUT*	67	F	Descending colon	Sarcoma-like	CD99	NA	NA
Case 34	Tamura et al. (30)	*MXD4::NUT*	34	F	Ovary	Sarcoma-like	Vimentin, CD99	Surgery and CT	Dead (9)
Case 35	Xu et al. (31)	*MXD4::NUT*	30	M	Penis	Sarcoma-like	NA	Surgery	Dead (48)

M, male; F, female; RCT, radiochemotherapy; RT, radiotherapy; CT, chemotherapy; NA, not available.

### Immunohistochemistry and EBER *in situ* hybridization

Four-micrometer sections underwent hematoxylin and eosin (H&E) staining and immunohistochemistry (IHC) using the following series of antibodies: NUT (clone C52B1, CST, Massachusetts, USA), PCK (clone AE1/AE3, BIO, Fujian, China), Cytokeratin 5/6 (CK5/6, clone D5/16B4, MXB, Fujian, China), Cytokeratin 7 (CK7, clone RN7, BIO), EMA (clone GP1.4, BIO), Leukocyte common antigen (LCA) (clone 2B11 and PD7/26, BIO), CD56 (clone UMAB83, BIO), synaptophysin (polyclonal, MXB), S-100 (clone 4C4.9, MXB), myogenin (clone EP162, BIO), desmin (clone MX046, MXB), P63 (clone UMAB4, BIO), CD34 (clone EP88, BIO), Ki-67 (clone MIB-1, DAKO, California, USA), and PD-L1 (clone SP263, BIO). Epstein–Barr virus (EBV) *in situ* hybridization (ISH) (EBER1/2 probes, BIO) was also performed according to the manufacturer’s instructions. Staining was performed on a Roche Ventana system. The evaluation of these staining procedures was performed as previously described (9).

### Fluorescence *in situ* hybridization

Fluorescence *in situ* hybridization (FISH) was performed using a *NUTM1* dual-color break-apart probe (Anbiping, Guangzhou, China) to assess *NUT* gene translocation according to the manufacturer’s instructions. The slides were observed under a ×100 objective magnification using a fluorescence microscope (Leica DM6000, Wetzlar, Germany) equipped with the Applied Imaging 4.0 analysis software (Genetix, England, UK). The case was considered translocation positive if more than 15% of the scored tumor cells (at least 100 cells) showed split signals as previously described (10, 11) (note: ALK FISH diagnostic criteria were followed). The slides were independently evaluated by two pathologists (W Wang and X Wu) with expertise in FISH analysis.

### Gene expression data analysis

RNA was extracted from tumor specimens using the RNeasy FFPE Kit (Qiagen, 73504, Hilden, Germany). The RNA sequencing method has been described previously (12, 13). Briefly, total RNA was extracted from tumor specimens using the RNeasy FFPE Kit (Qiagen, 73504) according to the manufacturer’s protocols. The NEBNext rRNA Depletion Kit (Human/Mouse/Rat) (NEB, #Z1955E, Massachusetts, USA) was chosen to remove the targeted ribosomal RNA (rRNA). After rRNA depletion and fragmentation, cDNA synthesis and next-generation sequencing (NGS) library preparation were performed using NEBNext^®^ Ultra™ II Directional RNA Library Prep Kit (NEB, E7760L). RNA-seq libraries were sequenced on the Gene + Seq-2000 with a paired-end 2 × 100 bp protocol, resulting in 20 Gb per sample. After removal of terminal adaptor sequences and low-quality data using fastp (version 1.3.1) and removal of rRNA reads by aligning clean reads to the rRNA database (download from NCBI) using bowtie2 (version 2.5.5), clean reads without known rRNA were aligned to the reference human genome (hg19) through STAR (version 2.7.11b). Fusions were detected using a customized version of Arriba (version v1.0.0) and annotated using in-house software annoFilterArriba (version v1.5.5) with the NCBI release 104 database using default parameters. The filtering criteria were as follows: 1) the supporting read threshold for considering a non-hotspot fusion positive was conservatively set at 10 or more supporting reads. (2) For all known fusions, two supporting reads were used as the positivity threshold (14). All final candidate fusions were manually verified using the Integrative Genomics Viewer browser. A series of quality control metrics were computed using RNA-SeQC (version 2.4.2) assessment. A threshold of ≥80 million mapped reads and ≥10 million junction reads per sample was set.

## Results

### Case 1

**Figure 1:**
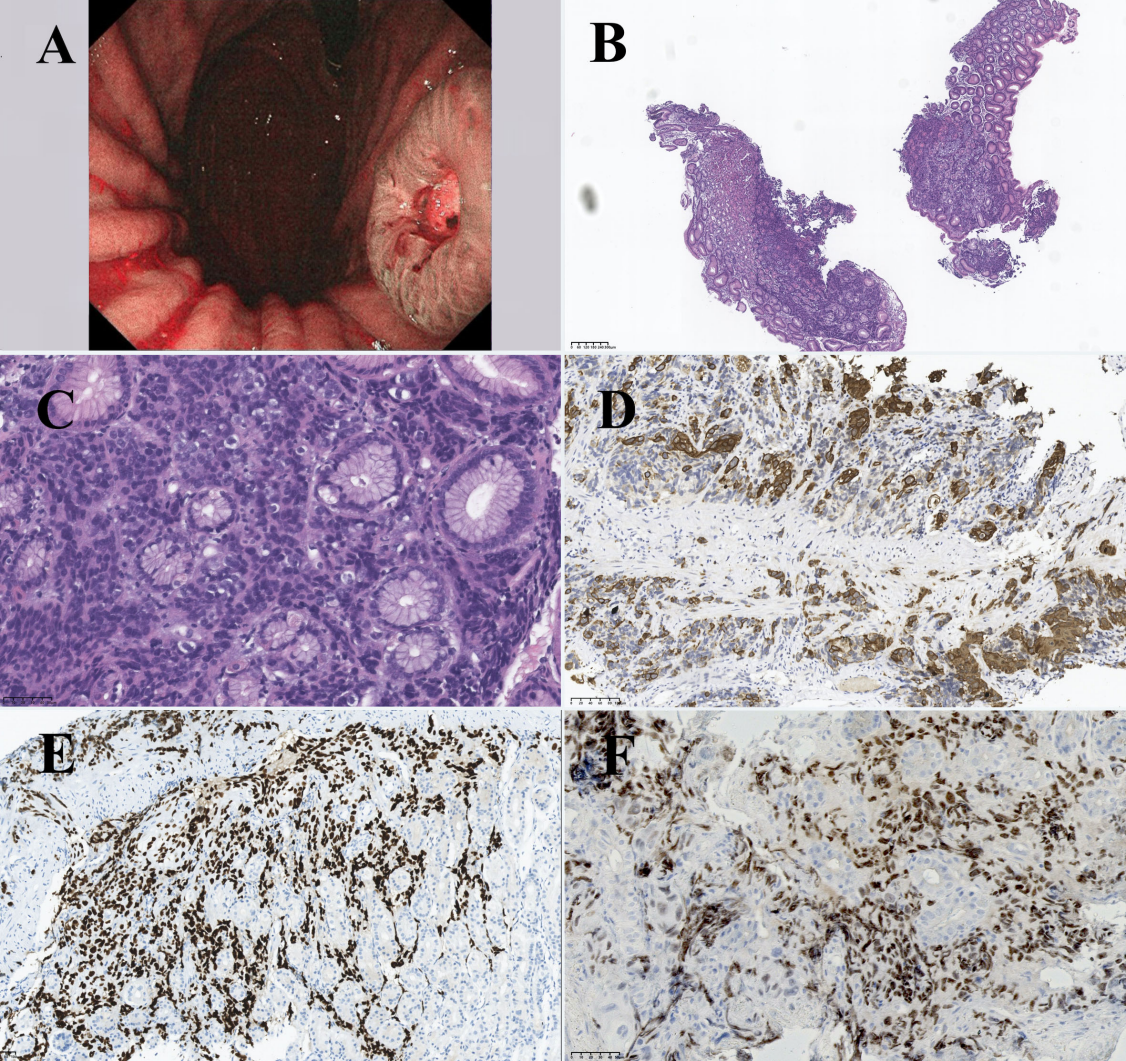
NC showing a small cell-like morphology (Case 1). Gastroscopy revealed a 1.2 cm ulcerative tumor in the gastric body **(A)**. The gastric mucosal glands were interspersed with extruded small cell-like tumor cells (**B**, HE x50). The nuclei of the tumor cells were extremely hyperchromatic with a high nucleocytoplasmic ratio (**C**, HE x400). The tumor cells were positive for PCK (**D** x200), P63 (**E** x200), and NUT antibodies (**F** x400).

**Figure 2:**
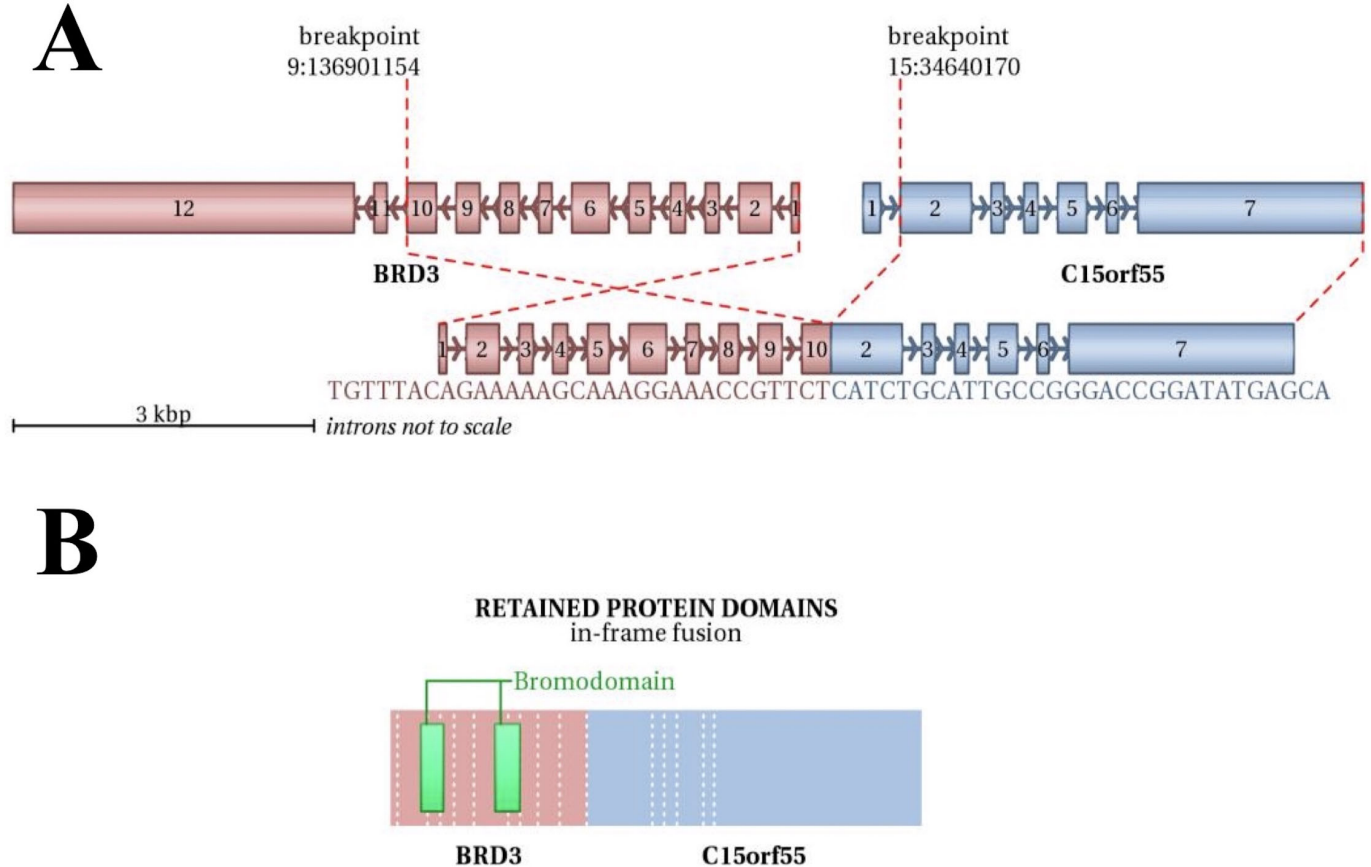
RNA sequencing of Case 1 revealed a gene rearrangement between the 3'-terminus of exon 10 of *BRD3* and the 5'-terminus of exon 2 of *NUTM1 (C15orf55)*, leading to abnormal expression of the cancer-associated *BRD3::NUTM1* fusion gene **(A)**. The bromodomain protein of BRD3 was retained **(B)**.

A 31-year-old male patient presented with generalized pain mainly in the large joints of the extremities, without an obvious pain-inducing event or factor. The patient also reported occasional headaches and abdominal distension. An X-ray finding demonstrated multiple osteolytic lesions in the vertebrae, pelvis, and other locations. Moreover, gastroscopy showed multiple ulcerative lesions in the greater curvature and anterior wall of the gastric body, with the largest being 1.2 cm in diameter (**Figure 1A**). Biopsy revealed diffusely distributed tumor cells with a small cell-like morphology, and poorly differentiated cancer cells were observed in some areas without obvious extrusion artifacts (**Figures 1B, C**). IHC was positive for PCK, EMA, P63, CK5/6, CK7, and NUT (**Figures 1D–F**). The Ki-67 index was 30%. RNA sequencing demonstrated that a chromosomal translocation t(9;15)(q34;q14) occurred between chromosomes 9 and 15 and produced the fusion oncogene *BRD3::NUTM1* (**Figure 2**).

Subsequently, the patient developed fatigue and had a poor appetite. Additionally, the patient lost weight and gradually lost the ability to stand, walk, or defecate. He received acupuncture treatment at the local hospital, but the symptoms and pain were not significantly relieved. Five months later, a computed tomography (CT) scan revealed thickening of the gastric antrum wall and multiple enlarged lymph nodes around the gastric antrum, hepatogastric ligament, and para-aortic region. Multiple bone destructions were observed throughout the body, accompanied by multiple soft tissue nodules appearing in the liver, pancreas, adrenal glands, greater omentum, parietal peritoneum, peritoneal cavity, and retroperitoneum. He received palliative care to manage the pain with limited effectiveness and was ultimately lost to follow-up in the fifth month.

### Case 2

**Figure 3:**
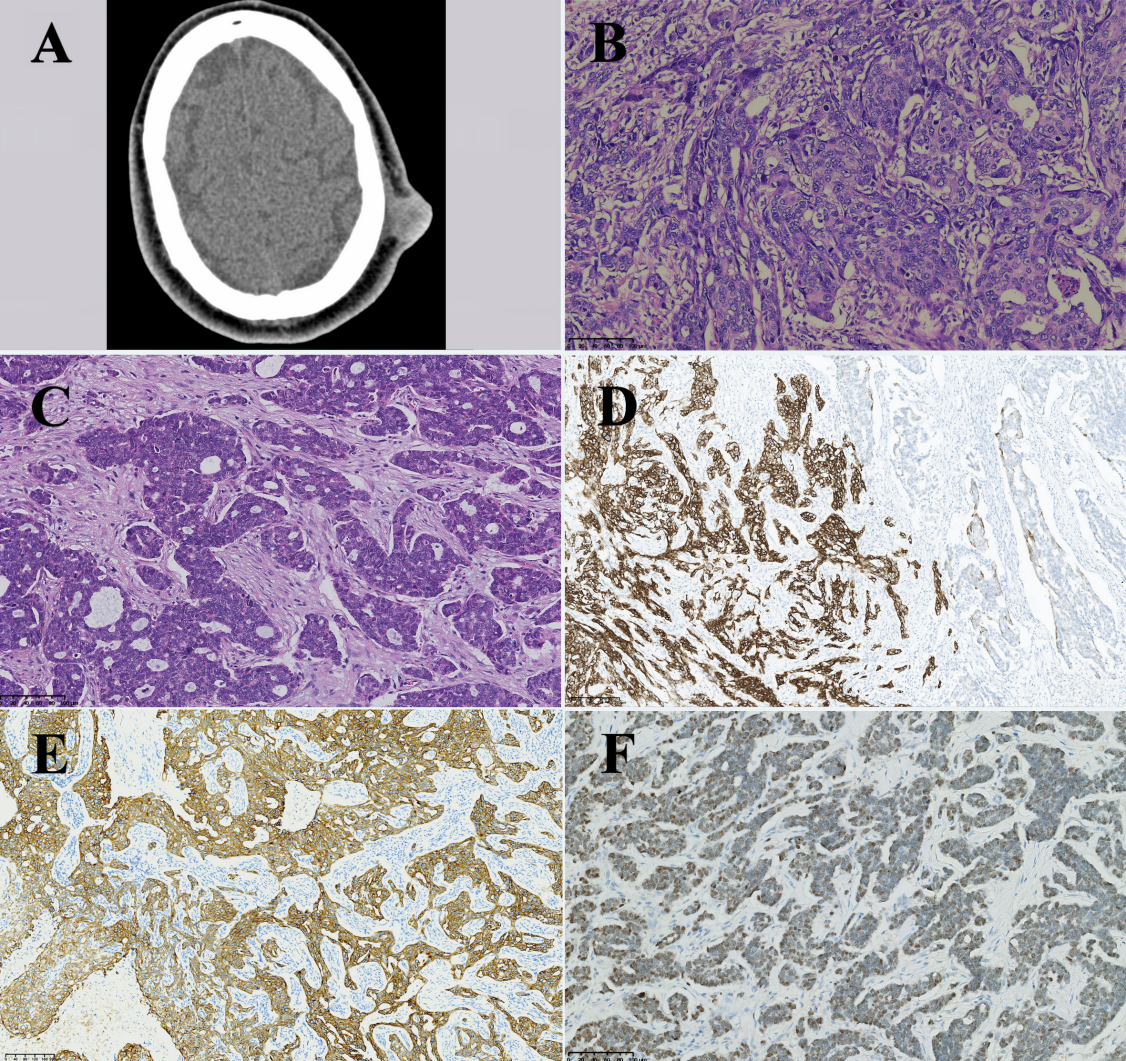
NC with glandular differentiation (Case 2, 3): CT revealed a 2.5 cm x 2.3 cm subcutaneous mass with uneven density and a clear boundary on the left calvaria vertex. The tumor corresponded to the thickening of the adjacent scalp **(A)**. Microscopically, the tumor appeared as a poorly differentiated carcinoma surrounded by fibrous stroma. Squamous (**B**, HE x200) and glandular differentiation (**C**, HE x200) could be observed. CK5/6 was positive in the squamous differentiated regions but negative in the glandular regions (**D** x100). Positive signals of CK7 (**E** x100) and NUT (**F** x200) were widely distributed.

A 60-year-old male patient presented with a gradually enlarging subcutaneous mass on the top of his head. The mass had a firm and darkened texture on the surface. CT imaging showed a 2.7 cm × 2.5 cm mass under the scalp (**Figure 3A**). Pathological examination revealed SCC accompanied by necrosis. Squamous differentiation and glandular differentiation were observed in focal areas. The tumor cells were positive for PCK, EMA, P63, CK5/6, CK7, and NUT, with a Ki-67 index of approximately 40% (**Figures 3B, D, E**). FISH analysis revealed a split signal in the *NUTM1* gene, and RNA sequencing demonstrated a chromosomal translocation t(3;15) (q25;q14) that led to the fusion of *WWTR1* and *NUTM1*. Subsequently, he underwent radiochemotherapy with regular follow-up.

Six months later, a CT scan revealed left cervical lymph node enlargement, which was further confirmed to be a metastasis after lymphadenectomy. One year later, he developed left ear pain with discharge and hearing loss. CT imaging revealed a solid mass with bone erosion behind the left auricle, which was surgically removed and confirmed to be a poorly differentiated SCC. Fourteen months later, a painful mass developed on his left neck, with PET–CT showing increased glucose metabolism in the left upper neck and parotid gland. SCC metastasis was suspected and subsequently confirmed through surgical resection. Two months later, a space-occupying lesion was identified in the right lower lobe on CT, and metastatic SCC was confirmed via fine-needle aspiration biopsy, with FISH indicating the existence of *NUTM1* rearrangement. Six months after lung biopsy, he died of tumor progression, 40 months after the initial diagnosis.

### Case 3

A 42-year-old male patient with no underlying disease presented with recurrent epistaxis for 3 months, with a bleeding volume of 50–100 mL each time. Nasopharyngoscopy revealed mucosal erosion in Little’s area of the nasal septum, which was covered with a small amount of blood clots. A poorly defined mass was observed in the left nasal cavity, accompanied by ipsilateral exophthalmos and cervical lymph node enlargement. CT imaging demonstrated a mass spanning the left nasal cavity, nasolacrimal duct, and inner canthus. No distant metastasis was found in organs such as the chest, liver, or brain. Histopathological examination of the biopsy revealed that the tumor was surrounded by abundant interstitial fibrosis, forming strips, cords, and nests. The malignant tumor cells were uniformly round- or oval-shaped with minimal cytoplasm. The nuclei of the tumor cells were irregular with thick nuclear membranes, vesicular chromatin, and prominent large acidophilic nucleoli. Glandular differentiation with intraluminal mucin was observed in the focal area (**Figure 3C**). No definitive squamous differentiation could be observed. Mitotic figures, neutrophil infiltration, and extensive necrosis were prominent within the tumor. IHC staining for PCK, CK5/6, CK7, and NUT (**Figure 3F**) was positive, with a Ki-67 proliferation index of 20%–40%. EMA IHC staining was positive at the luminal border. RNA sequencing revealed *BRD3::NUTM1* gene rearrangement (exon 10:exon 2), namely, a t(9;15)(q34;q14). Although timely and aggressive chemotherapy and radiotherapy were performed, the tumor progressed rapidly, and he died 5 months after the initial diagnosis.

In addition, negative results were obtained in all three cases for LCA, CD56, S-100, myogenin, desmin, CD34, PD-L1, and EBER *in situ* hybridization. FISH results for Case 2 and RNA sequencing results for all three cases are provided in **Supplementary Material**.

## Discussion

NC accounts for approximately 1.1% of poorly differentiated/undifferentiated carcinomas of the head and neck (15). The clinical course of NC is usually highly aggressive, with more than 80% of patients dying within the first year after initial diagnosis (16). NC is usually resistant to non-specific radiochemotherapy, but most patients who are diagnosed in a timely manner benefit significantly from various *NUT*-rearranged targeted therapies (17–19). Therefore, precise and rapid identification of NC is crucial. However, the diagnosis of NC poses a great challenge to pathologists. The histology of canonical NC appears to reveal poorly differentiated carcinoma with or without squamous differentiation, typically presenting small- to medium-sized basaloid cells with one to two prominent nucleoli. In some cases, separation artifacts and clear cytoplasm could appear between the cytoplasm and cell membrane, resulting in a fried egg-like appearance (1, 15). Occasionally, atypical morphologies such as rhabdomyoid (20), sarcomatoid (21), and chondroid (22) features could also be present. Given the substantial morphological heterogeneity of NC, some scholars have suggested that all poorly differentiated carcinomas occurring in the chest, head, and neck should be screened with NUT-IHC staining (1, 16, 23).

Interestingly, we noted that all three NC patients in our study harbored non-canonical *NUT* fusion types, with the lesions frequently occurring in rare locations and showing non-canonical morphological characteristics. IHC was used to exclude more common tumors at these sites, including neuroendocrine tumors, malignant melanoma, hematolymphoid tumors, mesenchymal tumors, and EBV-associated tumors. A literature review of NC patients with unusual clinicopathological features was therefore conducted (**Table 1**; some cases were not included due to canonical morphology or unavailable genetic testing results).

Consistent with our findings, NC with non-canonical fusion types occurred at rare sites and had atypical morphological features in many cases: consistent with Cases 2 and 3 in our cohort, glandular differentiated NC was observed in the inguinal subcutaneous tissue of patients with *BRD3::NUTM1* rearrangement (24). NCs with epithelioid or myoepithelioid morphology were observed in the masticator space (25), central nervous system, paravertebral body, epidural space, and lung (4), accompanied by a *CIC::NUTM1* rearrangement. Patients harboring *ZNF532::NUTM1* or *MXD1::NUTM1* fusion exhibited rhabdomyoid morphology in the mandible (20) or gastric wall (26), respectively. In addition, NCs with sarcomatoid morphology were also common in patients with *MGA::NUTM1* or *MXD4::NUTM1* fusion, occurring in the lung (3, 21), thigh (5, 27), dura mater (27), foot (28), pleura (29), chest wall (5), colon (3, 6), pelvis (5), ovary (30), and penile shaft (31). Thyroid-derived NCs often exhibit follicular architecture, colloid-like secretions, or even plasmacytoid morphology, with approximately 50% to 75% of them harboring *NSD3::NUTM1* rearrangement (32, 33). Dickson et al. reported that an arm tumor harboring *BCORL1::NUTM1* fusion displayed polymorphic morphology, including polygonal cells and spindle cells, with regions containing chrysanthemum-like structures and chondromyxoid matrix (26).

Traditionally, NC is considered to have an extremely bad prognosis, with a median survival of only 6–10 months (16, 34). Although some large cohort studies have shown no statistically significant difference in survival between patients with and without *BRD4::NUTM1* rearrangement (16, 35), several reports have documented significant individual variation in survival, with patients with non-canonical fusion types showing a better outcome (**Table 1**). French et al. reported that the survival time of patients with non-*BRD4::NUTM1*-rearranged NC was nearly four times longer than that of patients with *BRD4::NUTM1*-rearranged NC (36). Chau et al. reported that chest NC patients with *BRD3::NUTM1* or *NSD3::NUTM1* rearrangement had better outcomes than those with *BRD4::NUTM1* rearrangement (37). In addition, thyroid NC, characterized by a high frequency of *NSD3::NUTM1* rearrangement, had a significantly better prognosis than other anatomical locations [5-year overall survival (OS) up to 58%], in which patients with NC-like morphology showed significantly worse OS than those with non-NC-like morphology (32). Scattered case reports further supported this tendency as follows. Dickson et al. reported a gastric NC patient with *MXD1::NUTM1* fusion demonstrating a 9-year survival period (26). Wangsiricharoen et al. (5) and Diolaiti et al. (27) reported that *MGA::NUTM1* fusion tumor patients exhibited an OS of 11 and 15 years, respectively, in the thigh and 7 years in the pelvis. Nishimura et al. reported a case of inguinal subcutaneous *BRD3::NUTM1* fusion with long-term survival (11 years) after the initial diagnosis (24). The second NC patient (Case 2) in our study with *WWTR1::NUTM1* rearrangement also survived for 40 months after the initial diagnosis. The survival time of patients with these unusual fusion types of NC varied significantly, as shown in **Table 1**, from death within 1 month after diagnosis to long-term survival. Furthermore, some reports have suggested that non-*BRD4::NUTM1*-rearranged NCs may not be sensitive to bromodomain inhibitors, a currently available NUT-targeted therapeutic agent (3). Therefore, in addition to IHC and FISH, NGS or RT-PCR may help identify specific fusion partners, which could provide valuable insight into tumor behavior and potentially inform therapeutic selection.

Based on the clinicopathological features of our cohort and the literature review above, it seems that the histological morphology and prognosis of NC appear to be associated with the fusion partner of the *NUTM1* gene to a certain extent. According to the literature, non-*BRD4::NUTM1* fusions were associated with distinct histologic phenotypes depending on the specific fusion type (e.g., *CIC*-, *MGA*-, *NSD3*-, and *MXD4*-associated fusions), while rare fusions (*WWTR1*, *ATXN1*, *MXD1*, etc.) were often associated with an extremely atypical location, histological morphology, and clinical course. If the grouping of NC patients can be based on the fusion partners, we speculate that the differences in treatment efficacy and prognosis may become more apparent. Unfortunately, case reports of NC with rare fusion types to date are still limited, so more NC reports are needed to investigate its detailed clinicopathological spectrum.

**Figure 4:**
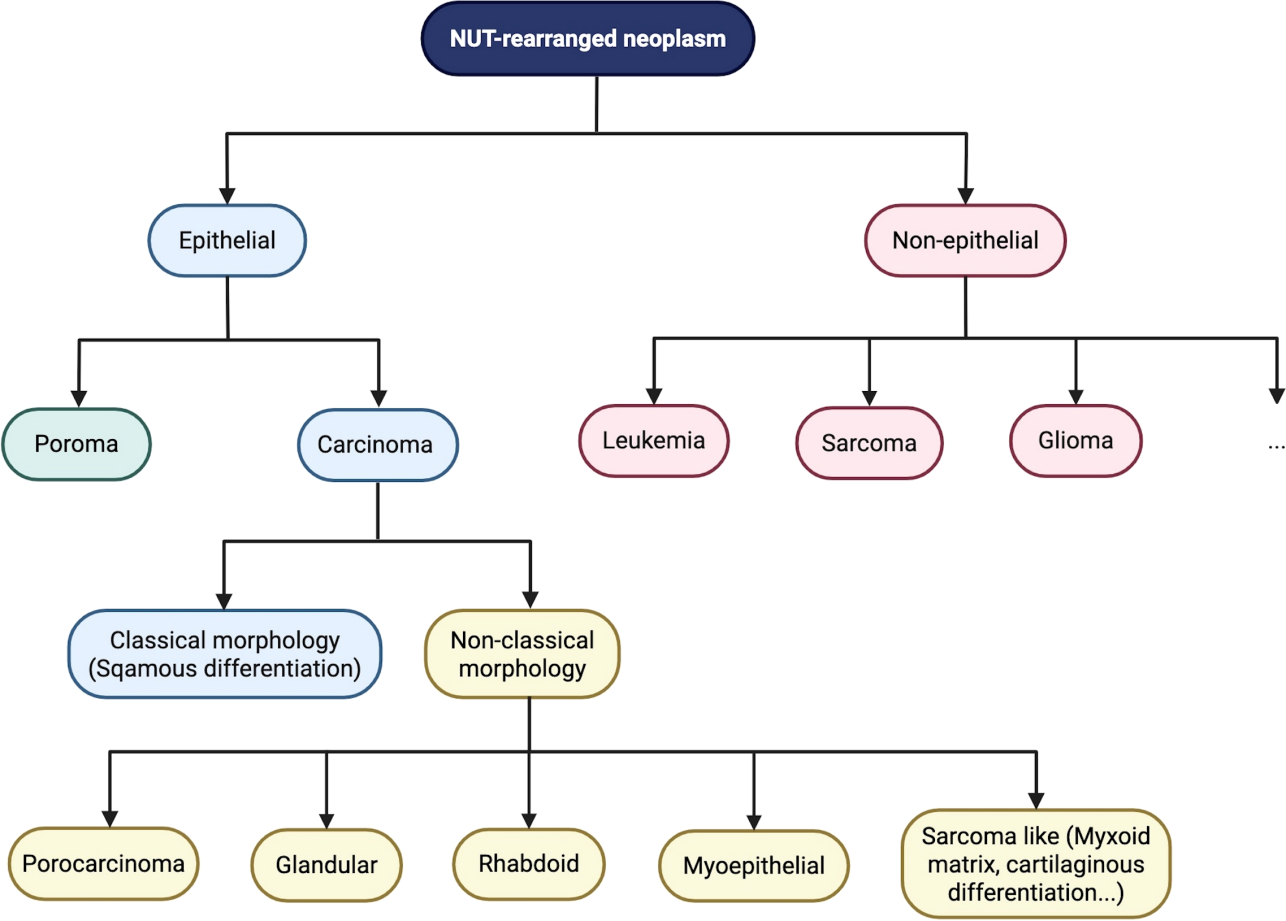
The histological types and morphological descriptions of NUT-rearranged neoplasms according to the literature.

In recent years, with the widespread use of NGS, *NUTM1* rearrangement has also been detected in tumors other than traditional NUT “carcinoma”, especially in skin adnexal tumors (7, 8, 38–44) (**Table 2**). In a study of 67 *NUT*-rearranged poroid neoplasms, 64 patients (95.5%) harbored *YAP1::NUTM1* fusion, two harbored *WWTR1::NUTM1* fusion (7, 44), and one harbored *EMC7::NUTM1* fusion (8). Notably, among the 67 poroid neoplasms, 27 were confirmed to be benign poroma, 26 (96.3%) harbored *YAP1::NUTM1* rearrangement, and one harbored *WWTR1::NUTM1* rearrangement (*WWTR1::NUTM1* fusion was also detected via RNA sequencing in Case 2 of our cohort). Furthermore, *NUTM1* rearrangement has also been detected in non-epithelial tumors, including sarcoma, acute lymphoblastic leukemia, and glioma (3, 45–48) (**Figure 4**). In these studies, the terms “*NUTM1*-rearranged neoplasm” (3, 45, 49) and “NUT sarcoma” (15, 28) were used to redefine these cases, which led to the following new proposition: does *NUTM1* rearrangement, like *BRAF* mutation, *ALK* rearrangement, or *NTRK* rearrangement, act as a driver gene and widely exist in human tumors of various origins, even benign tumors?

**Table 2:** Summary of current reported cases of *NUT-*rearranged cutaneous poroid neoplasms in the literature.

Author	Age (years)	Sex	Location	Fusion partner/diagnosis			Outcome (months)
				*YAP1*	*WWTR1*	*EMC7*	
Hartsough et al. (38)	55	M	Scrotum	1 porocarcinoma			Alive (4)
Parra et al. (39)	81	F	Foot	1 porocarcinoma			NA
Christensen et al. (40)	51	F	Scalp	1 porocarcinoma			Alive (25)
Agaimy et al. (41)	28 82	2 M	External auditory canal	2 porocarcinoma			Alive (12) Alive (6)
Sekine et al. (7)	NA	NA	Head and neck, trunk, extremity	21 poroma 6 porocarcinoma	1 poroma		NA
Russell-Goldman et al. (42)	NA	NA	NA	5 porocarcinoma			NA
Prieto-Granada et al. (43)	50–83	3 M 2 F	Forehead, thigh, foot, knee	2 poroma 3 porocarcinoma			NA
Macagno et al. (8)	NA	NA	NA	12 poroid neoplasm		1 poroid hidradenocarcinoma	NA
Snow et al. (44)	13–90	6 M 5 F	Abdomen, back, elbow, foot, knee, nose, temple	3 poroma 6 porocarcinoma 1 dermal duct tumor	1 poroma with porocarcinoma		NA

M, male; F, female; NA, not available.

Canonical NC is predominantly an aggressive, poorly differentiated carcinoma arising from midline structures such as the thorax, head, and neck. Our study indicates that *NUTM1*-rearranged tumors occurring at rare locations are distinct from canonical NC and may require a different diagnostic framework. Case 2 particularly highlights this uncertainty: a calvaria vertex lesion with *WWTR1::NUTM1* fusion showing glandular differentiation and a 40-month survival period. Therefore, not all *NUTM1*-rearranged tumors should be equated with canonical NC.

For tumors arising outside canonical midline sites, we recommend integrating histomorphology (e.g., glandular, poroid, or myoepithelial features), immunophenotype, and clinical behavior with molecular testing results. Before more explicit diagnostic criteria are established, the use of descriptive terms such as “*NUTM1*-rearranged tumor” with site and morphological modifiers may be considered. More cases of non-canonical NC need to be accumulated in the future to refine diagnostic criteria and construct a more unified conceptual framework for *NUTM1*-rearranged tumors.

In conclusion, after reviewing the canonical NC represented by *BRD4::NUTM1*, our study focused on reporting and summarizing NC cases with non-canonical fusion variants, characterized by uncommon anatomical sites and distinctive histopathological morphology. We aim to help pathologists quickly and accurately identify and diagnose these non-canonical NC cases. When diagnosing and classifying *NUT*-rearranged malignant tumors, it is crucial to preliminarily classify them into carcinoma or sarcoma based on histomorphology and immunophenotype and to further classify them into canonical or non-canonical fusion subtypes, which could potentially inform treatment decisions with available targeted agents. Therefore, additional case reports and larger studies are needed to better understand the impact of different *NUTM1*-rearranged tumors on downstream pathways and biological behavior, thereby guiding drug development and improving the prognosis of NC.

## Data Availability

The original contributions presented in the study are publicly available. This data can be found here: https://ddbj.nig.ac.jp/search/, accession numbers SAMD01928453, SAMD01928454, and SAMD01928455.
