# Supplementary material for: Expanding the spectrum of non-canonical NUT carcinoma: clinicopathological and molecular characterization of BRD3::NUTM1 and WWTR1::NUTM1 fusion variants
Source: Front Oncol. 2026 Jul 1;16:1828484. doi: 10.3389/fonc.2026.1828484 (PMC13368637; doi:10.3389/fonc.2026.1828484)

| Case | Fusion Gene | Transcript | Functional Region | Cytogenetic Annotation |
| --- | --- | --- | --- | --- |
| 1 | *BRD3::C15orf55* | NM_007371.3/NM_175741.1 | EX10:EX2 | t(9;15)(q34;q14) |
| 2 | *WWTR1::C15orf55* | NM_015472.4/NM_175741.1 | EX3:EX2 | t(3;15)(q25;q14) |
| 3 | *BRD3::C15orf55* | NM_007371.3/NM_175741.1 | EX9:EX4 | t(9;15)(q34;q14) |

**FISH result (Case 2):**


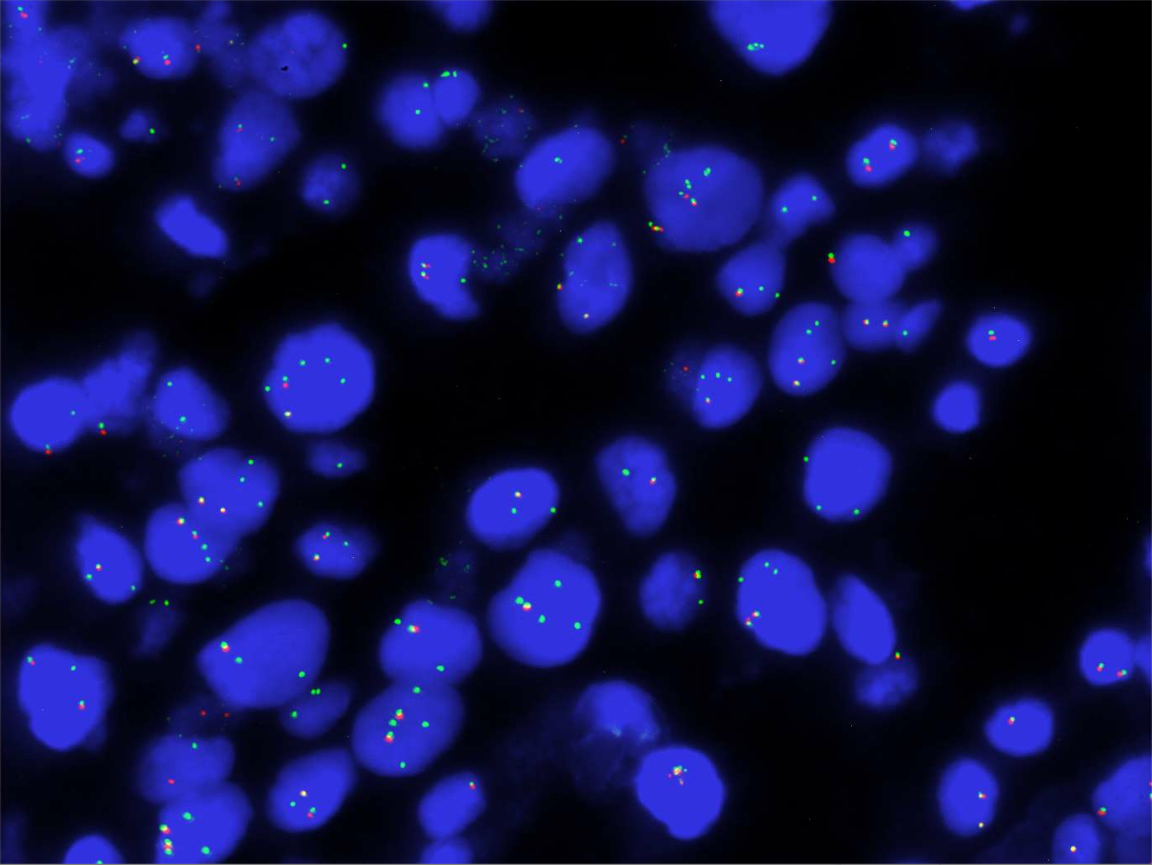


**The Integrative genomics viewer (IGV) map of RNA-seq reads.**

Case 1:


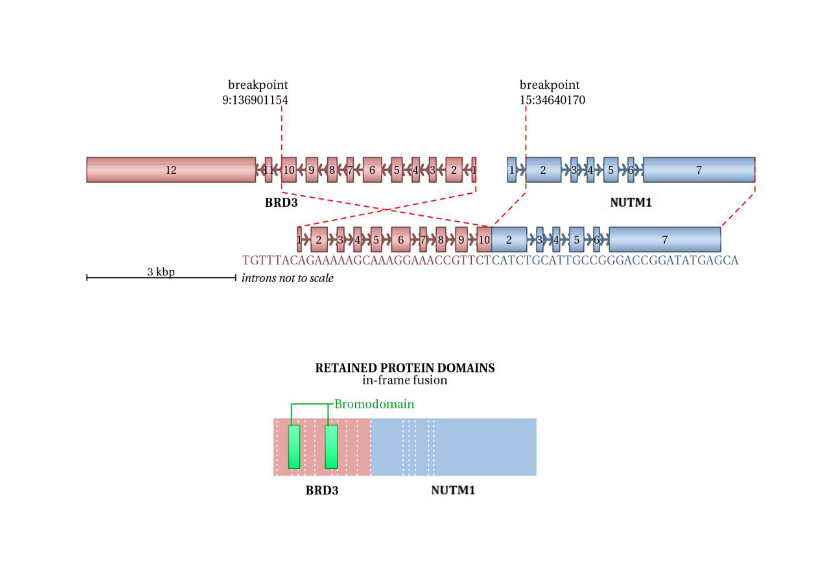


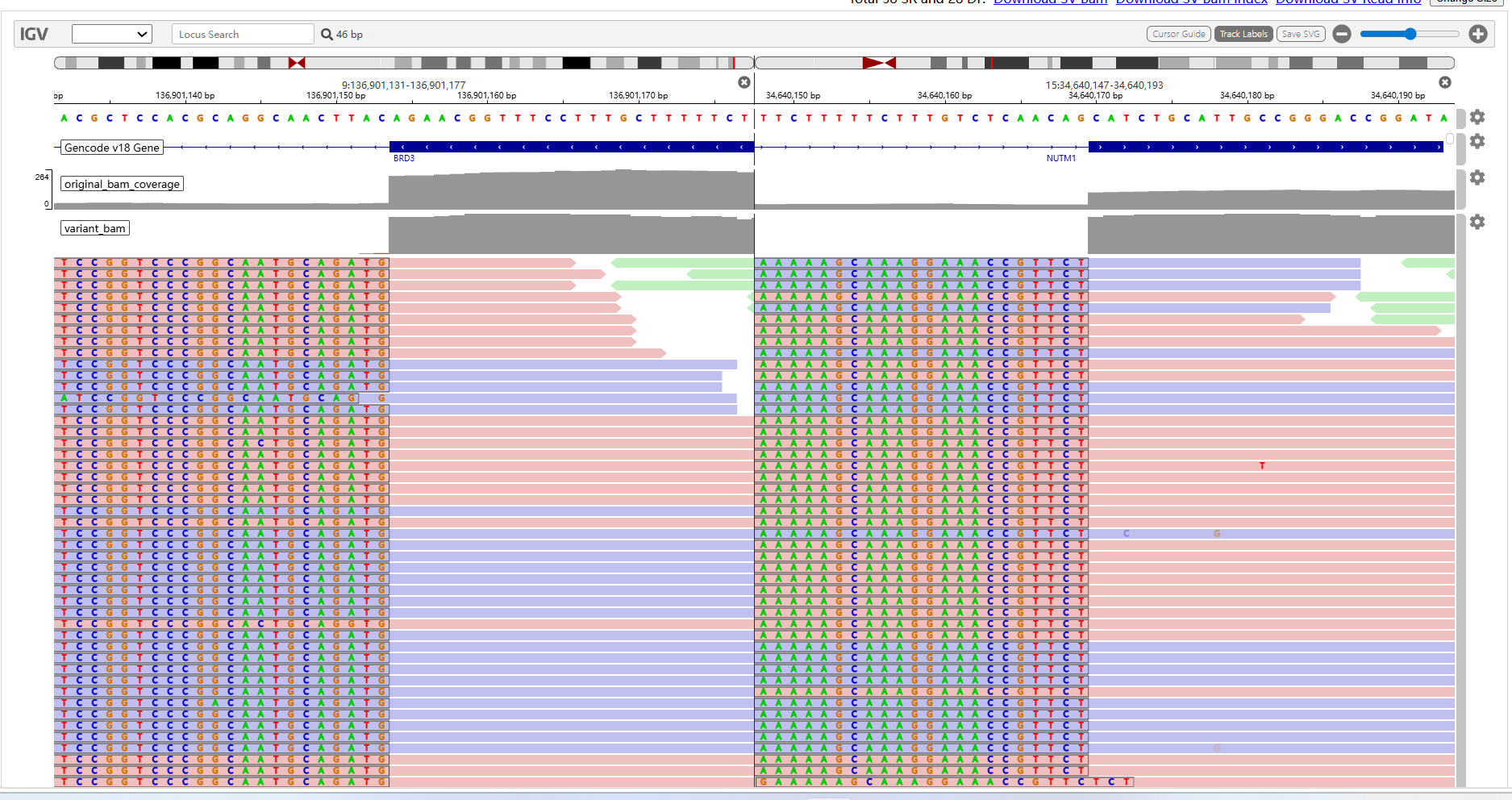


Case 2:


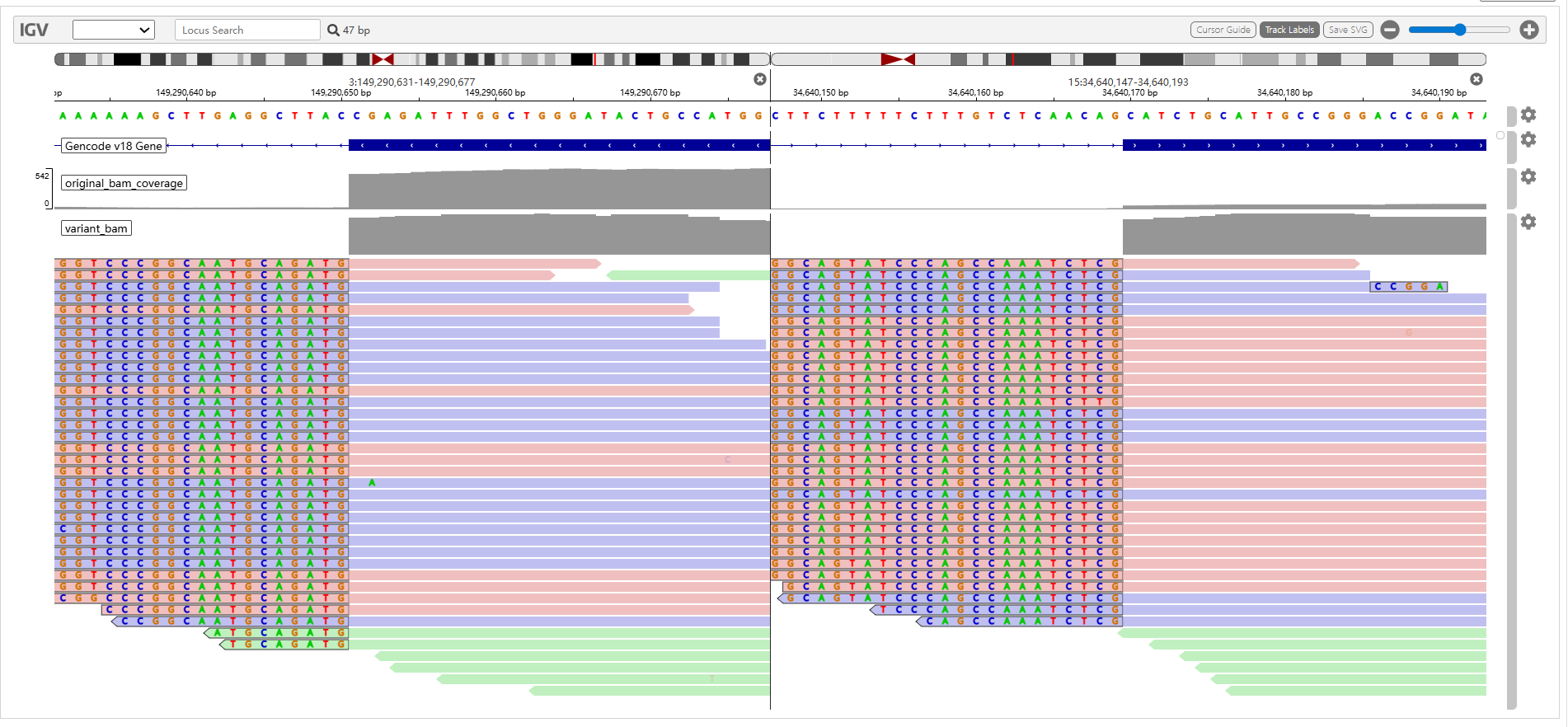


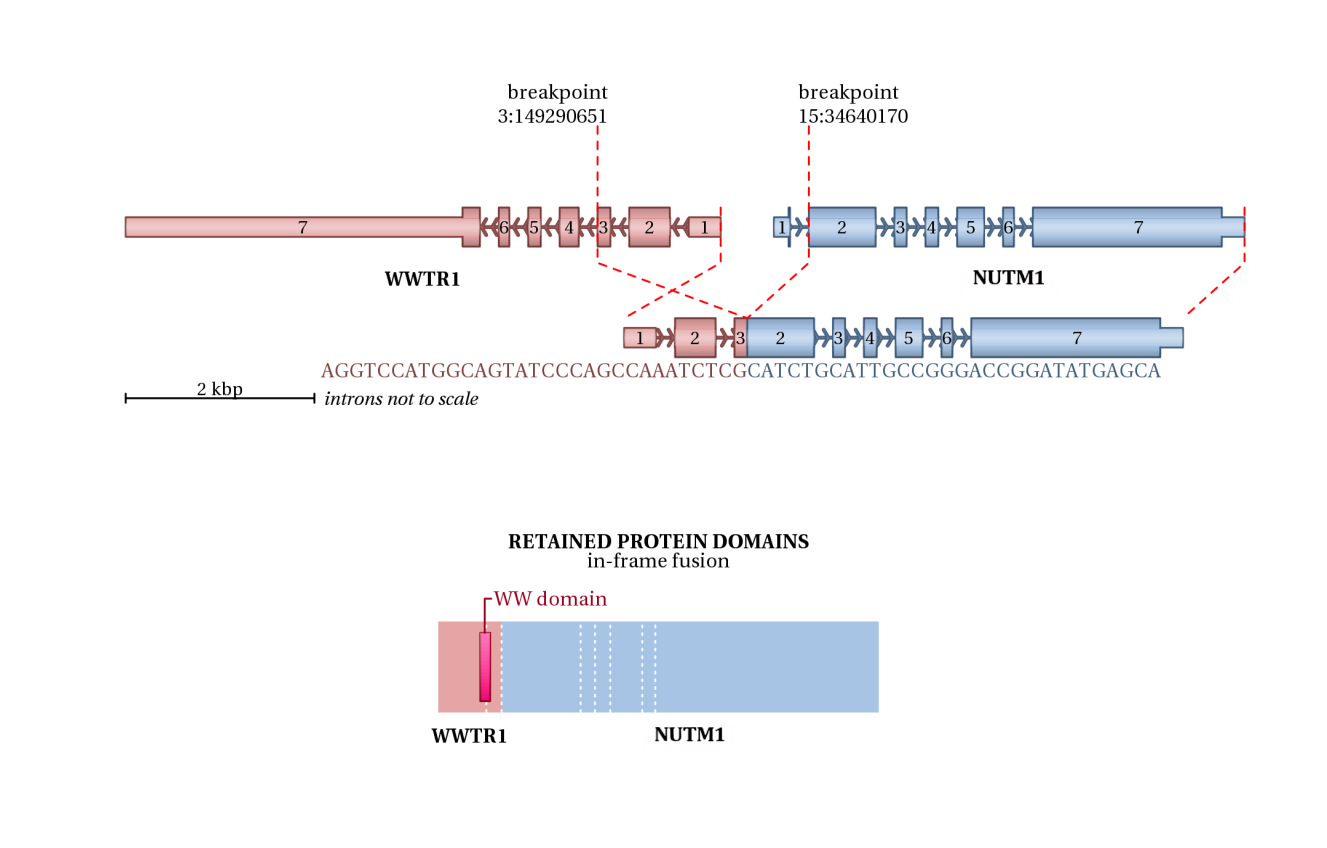


Case 3:


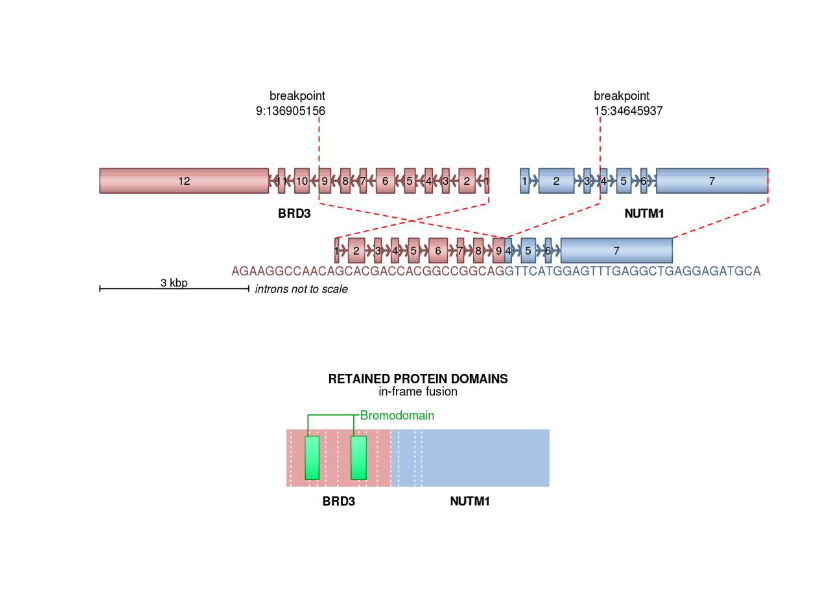


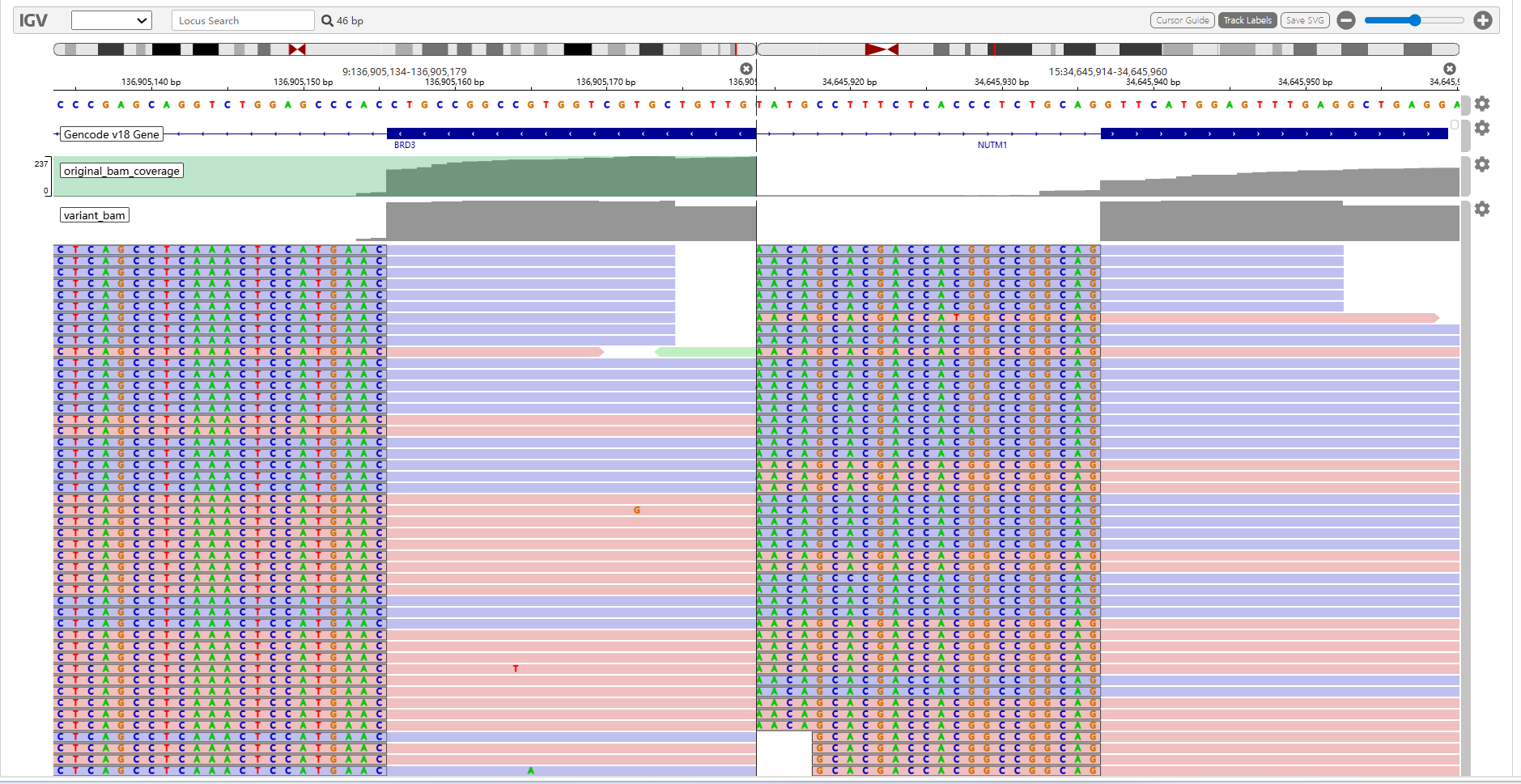

Supplement: Supplementary file 1 [file DataSheet1.docx]
